# Amyloid-β peptides slightly affect lifespan or antimicrobial peptide gene expression in *Drosophila melanogaster*

**DOI:** 10.1186/s12863-020-00866-y

**Published:** 2020-10-22

**Authors:** Mikhail V. Shaposhnikov, Nadezhda V. Zemskaya, Lyubov А. Koval, Natalya R. Minnikhanova, Olga I. Kechko, Vladimir A. Mitkevich, Alexander A. Makarov, Alexey А. Moskalev

**Affiliations:** 1 Engelhardt Institute of Molecular Biology, Russian Academy of Sciences, 119991 Moscow, Russia; 2 Institute of Biology of Komi Science Center of Ural Branch of RAS, 167982 Syktyvkar, Russia

**Keywords:** Lifespan, Aging, *Drosophila melanogaster*, Amyloid-β peptides, Antimicrobial peptides, Transcription factor FOXO, Peroxiredoxin 5

## Abstract

**Background:**

Beta-amyloid peptide (Aβ) is the key protein in the pathogenesis of Alzheimer’s disease, the most common age-related neurodegenerative disorder in humans. Aβ peptide induced pathological phenotypes in different model organisms include neurodegeneration and lifespan decrease. However, recent experimental evidence suggests that Aβ may utilize oligomerization and fibrillization to function as an antimicrobial peptide (AMP), and protect the host from infections. We used the power of *Drosophila* model to study mechanisms underlying a dual role for Aβ peptides.

**Results:**

We investigated the effects of *Drosophila* treatment with three Aβ42 peptide isoforms, which differ in their ability to form oligomers and aggregates on the lifespan, locomotor activity and AMP genes expression*.* Aβ42 slightly decreased female’s median lifespan (by 4.5%), but the effect was not related to the toxicity of peptide isoform. The lifespan and relative levels of AMP gene expression in male flies as well as locomotor activity in both sexes were largely unaffected by Aβ42 peptide treatment. Regardless of the effects on lifespan, Aβ42 peptide treatment induced decrease in AMP genes expression in females, but the effects were not robust.

**Conclusions:**

The results demonstrate that chronic treatment with Aβ42 peptides does not drastically affect fly aging or immunity.

## Background

Traditionally beta-amyloid peptide (Aβ) considered as the key protein in pathology of Alzheimer’s disease (AD), the most common inflammatory neurodegenerative disease in humans [1, 2]. The accumulation and deposition of insoluble, aggregated Aβ peptides in extracellular amyloid plaques in the brain is one of the pathological hallmarks of AD [3]. The soluble Aβ oligomers act as active neurotoxins, causing neuronal dysfunction, loss of synaptic connections, cell death, and subsequent detrimental events of AD [4, 5].

However, a growing body of evidence suggests that Aβ can also possesses physiological roles [6, 7]. Particularly Aβ may function as an antimicrobial peptide (AMP), а component of the innate immune system [8, 9]. Aβ utilizes oligomerization and fibrillization to protect the host from a broad spectrum of infectious agents including protozoans, fungi, bacteria, mycobacteria, and enveloped viruses [7, 8].

The fly model allows using the power of *D. melanogaster* genetics to identify mechanisms underlying the effects of exogenous β-amyloid peptides [10, 11]. Although endogenous Aβ peptides are not produced normally in *Drosophila* [12], neurodegenerative phenotypes induced by the exogenous Aβ peptides in *Drosophila* suggest a conserved function [10, 11]. Overexpression of human Aβ42 peptides in the nervous system of the fly results in phenotypes associated with neuronal degeneration [10, 13], locomotor decline [14, 15] and a lifespan decrease [16]. No positive effects of exogenous Aβ peptides in *Drosophila* model was published to date. However, since the microbial quantity is a predictor of fly longevity [17], we assume that the benefits of exogenous Aβ may be possible because of its antimicrobial activity.

Previous studies have shown that constitutive ubiquitous overexpression of anti-microbial peptide gene *Diptericin* is sufficient to increase antioxidant enzyme activities and tolerance to hyperoxia [18]. Conditional (RU486-mediated) activation of ubiquitous or gut specific overexpression of single AMP genes *Drosocin* and *CecropinA1* in adult flies leads to reduced immune challenge or intestinal stress response, improved intestinal integrity and lifespan [19]. We previously showed that the lifespan extending effect of pectins is associated with activation of expression of the NF-κB-dependent AMP genes *Defensin*, *Drosomycin* and *Metchnikowin* [20]. At the same time constitutive ubiquitous or fat body specific activation of expression of several different classes of Relish target AMPs (including *Attacin A*, *Attacin C*, *Attacin D*, *Cecropin A1*, *Defensin*, *Diptericin*, *Drosocin*, *Drosomycin*, and *Metchnikowin*) or overexpression of some individual AMP genes (including *Attacin A*, *Cecropin A1*, *Defensin*, and *Metchnikowin*, but not *Drosocin* and *Drosomycin*) induced cytotoxic effects and significantly shortened lifespan [21]. An earlier analysis of the aging-associated changes in the transcriptome revealed a significant increase in the level of expression of AMP genes in aging flies [22, 23]. It was also noted that the level of AMPs expression in young flies correlates negatively with lifespan [23]. Underexpression of Relish in the fat body beginning in the second half of lifespan prevented age-related overactivation of AMPs and extended longevity [21]. We also found that life-long pharmacological inhibition of NF-κB activity increases *Drosophila* lifespan [24]. Numerous studies demonstrated that stimulation of AMPs production by activation of upstream components of the innate immunity cell signaling pathways, such as peptidoglycan recognition protein (PGRP-LE) [25] or suppressing negative regulators, such as *dnr1* [26], or *pirk*, *trbd*, and *tg* [27], lead to proinflammatory state, neurodegeneration, and shortened lifespan. Thus, AMPs as well as Aβ peptides can be either harmful or protective in different model systems and experimental conditions.

The aim of this work was to investigate the effects of exogenous amyloid-β peptides on *Drosophila* lifespan and locomotor activity. Since Aβ peptides has AMP activity and treatment with Aß would be expected to influence infection rates, we analyzed the mRNA level of the antimicrobial peptide genes. In this study, we used 3 amyloid-β peptide isoforms, associated with human AD, including Aβ42 (non-modified Aβ, one of the main variants associated with familial forms of AD), isoD7-Aβ42 (Aβ42 peptide variant with isomerized Asp7, one of the most abundant age-related Aβ species within amyloid plaques) [28, 29], and pS8-Aβ42 (phosphorylated variant of Aβ42 with increased ability to form toxic aggregates as compared with Aβ42, involved in the pathogenesis of the most common sporadic form of AD) [30]. We revealed that Aβ42 and pS8-Aβ42, but not more toxic Aβ42 form isoD7-Aβ42, induced minor decline of female’s median lifespan while lifespan in male flies and locomotor activity in both sexes were not affected. The expression level of AMP genes (*CecropinA1*, *Defensin*, *Drosocin*, *Drosomycin*, and *Metchnikowin*) in males and females was slightly changed regardless of the effects on lifespan. The obtained results suggest that chronic treatment with Aβ42 weakly affects *Drosophila* aging and immunity.

## Results

### Lifespan and locomotor activity

**Fig. 1:**
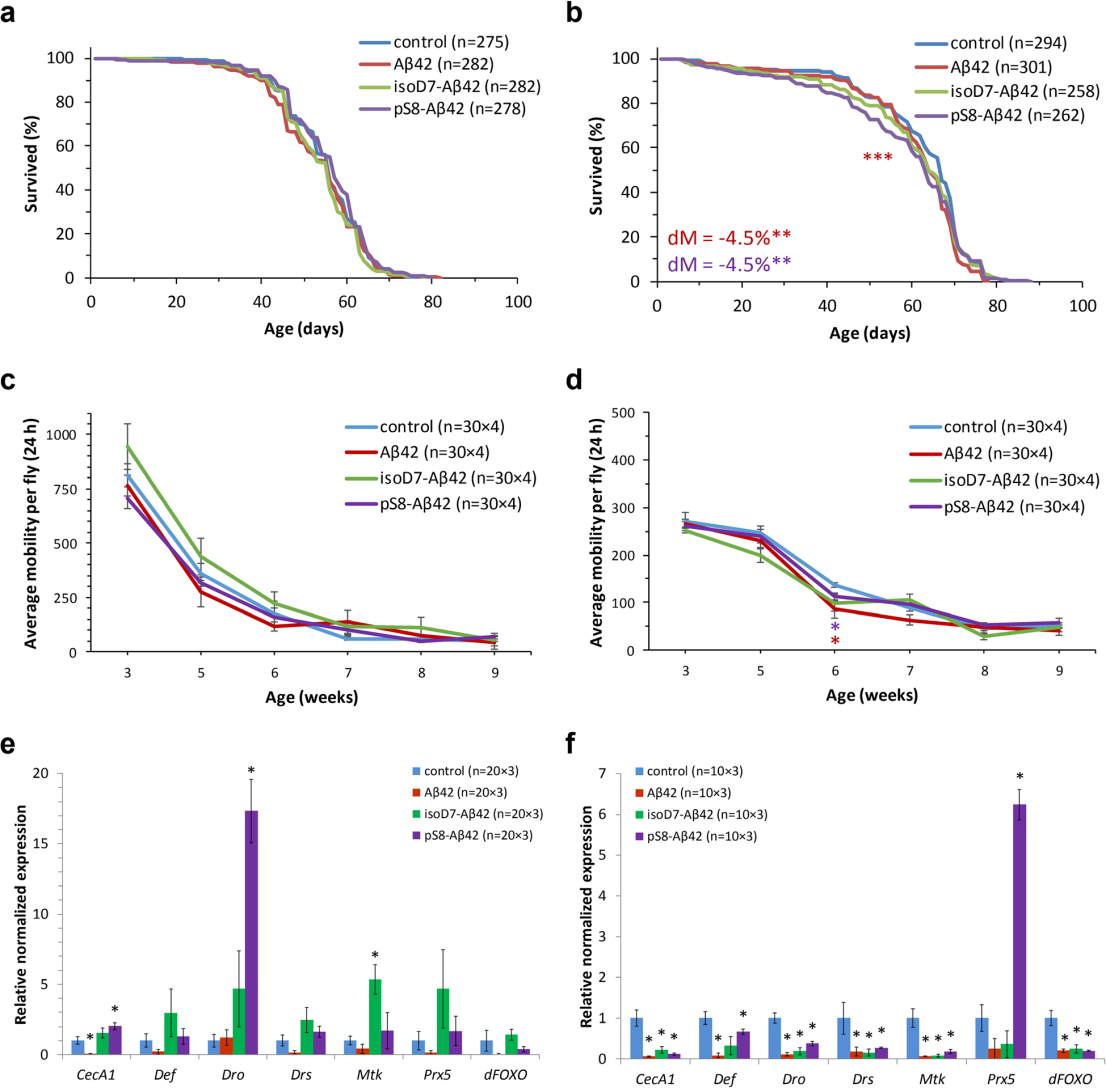
Effects of the amyloid-β peptides on lifespan (**a**, **b**), locomotor activity (**c**, **d**), and gene expression (**e**, **f**) in males (**a**, **c**, **e**) and females (**b**, **d**, **f**). *р < 0.05, **р < 0.01, ***p < 0.001, log-rank test (survival data comparison), Fisher’s exact test (median lifespan (dM) comparison), and Student’s t-test (comparison of locomotor activity and gene expression). The error bars show standard errors. For lifespan analysis the total number of flies (n) used in two replicates (5 vials in each) is indicated in parentheses. For locomotor activity and qRT-PCR analyses the number of flies (n) used in each replication of experiment multiplied by the number of replicates are indicated in parentheses. For detailed description see Materials and methods

The statistically significant effects of Aβ42, isoD7-Aβ42, and pS8-Aβ423 on male’s lifespan were not detected (*p* > 0.05, Fisher’s exact and log-rank tests) (Table 1, Fig. 1a). Using Cox proportional hazards analysis (Table 2) we found that in Aβ42 and isoD7-Aβ42 male variants there was an elevated hazard ratio (risk of death) of vial covariates (*p* < 0.01, likelihood ratio test), while effects of Aβ treatment remained insignificant (*p* > 0.05, likelihood ratio test). It was found that Aβ42 and pS8-Aβ42, but not isoD7-Aβ42 caused a statistically significant decrease in the median lifespan of females by 4.5% (*p* < 0.01, Fisher’s exact test) (Table 1, Fig. 1b). The hazard ratio of the females treated with Aβ42 peptide was slightly (1.201 times) increased (*p* < 0.05, likelihood ratio test) compared to those of the control flies (Table 2).

**Table 1:** Influence of Aβ peptides on median and maximum lifespan

Variant	Sex	M (days)	dM (%)	FET (p)	90% (days)	d90% (%)	WAT (p)	n
control	male	56			66			275
Aβ42	male	56	0.0	0.4706	66	0.0	0.464	282
isoD7-Aβ42	male	56	0.0	0.3906	64	−3.0	0.053	282
pS8-Aβ42	male	56	0.0	0.7325	64	−3.0	0.293	278
control	female	67			74			294
Aβ42	female	64	−4.5*	0.0024	71	−4.1	0.078	301
isoD7-Aβ42	female	64	−4.5	0.1668	74	0.0	0.554	258
pS8-Aβ42	female	64	−4.5*	0.0022	74	0.0	0.995	262

M (days) - median lifespan; 90% (days) - age of 90% mortality (maximum lifespan); dM (%), d90% (%), − differences between median and maximum (age of 90% mortality) lifespan of control and experimental flies, respectively; n - number of flies; *p < 0.01, FET - Fisher’s exact test (median lifespan comparison), WAT - Boschloo’s (Wang-Allison) test (maximum lifespan comparison)

**Table 2:** Cox proportional hazards analysis. Proportional hazard modeled for Aβ peptide (treatment versus control) and vials (10 vials in each experimental variant) as covariates with partial likelihood estimation

Variant	Risk factor	HR	SE	*P*-value
Aβ42 males	vial	**1.048**	0.015	**0.001**
	Aβ peptide	0.924	0.085	0.355
Aβ42 females	vial	0.971	0.029	0.313
	Aβ peptide	**1.201**	0.083	**0.028**
isoD7-Aβ42 males	vial	**1.054**	0.015	**0.001**
	Aβ peptide	1.008	0.043	0.848
isoD7-Aβ42 females	vial	0.971	0.029	0.313
	Aβ peptide	0.993	0.043	0.861
pS8-Aβ42 males	vial	1.005	0.004	0.159
	Aβ peptide	0.961	0.029	0.179
pS8-Aβ42 females	vial	0.971	0.029	0.313
	Aβ peptide	1.013	0.028	0.643

Hazard ratios (HR) indicates fold change of the hazard (risk of death) for variants with higher values of that variable. The risk factor Aβ peptide is encoded by 1 (untreated control) and 2 (treated with Aβ peptide). The vials are numbered from 1 to 10 in each experimental variant as pseudoreplicates. HR greater than one indicates increased risk of death. Significant estimates in bold. SE - standard error

In addition to the lifespan, the spontaneous locomotor activity was used for Aβ toxicity analysis (Fig. 1c and d). Locomotor activity of Aβ treated males was unaffected (Fig. 1c). Despite locomotor activity of Aβ42 (by 36.8%) and pS8-Aβ42 (by 18.0%) treated females at the age of 6 weeks showed decrease compared with control untreated females (*p* < 0.01, Student’s t-test) (Fig. 1d) ANOVA revealed that there is no a statistically significant difference between the control and Aβ treated animals (*p* > 0.05, Source of variation: Conditions) (Table 3). At the same time by using the ANOVA, we showed a significant difference in movement capacity between male and female flies of different ages in control and experimental variants (*p* < 0.001, Source of variation: Age) (Table 3).

**Table 3:** Analysis of differences in locomotor activity factored by age and conditions using two-way ANOVA

Variant	Source of variation	SS	DF	MS	F	*P*-value	F-crit
males							
pS8-Aβ42	Condition	4231.89	1	4231.89	0.918	0.344	7.396
	**Age**	3,031,998.79	5	606,399.76	131.572	**4.4e-22**	3.574
	Interaction	25,648.17	5	5129.63	1.113	0.371	3.574
	Within (errors)	165,919.81	36	4608.88			
	Total	3,227,798.65	47				
Aβ42	Condition	3615.74	1	3615.74	0.623	0.435	7.396
	**Age**	3,237,235.33	5	647,447.07	111.585	**7.1e-21**	3.574
	Interaction	34,015.75	5	6803.15	1.172	0.342	3.574
	Within (errors)	208,882.47	36	5802.29			
	Total	3,483,749.29	47				
isoD7-Aβ42	**Condition**	46,787.54	1	46,787.54	4.357	**0.044**	7.396
	**Age**	4,055,146.09	5	811,029.22	75.526	**4.6e-18**	3.574
	Interaction	17,717.56	5	3543.51	0.33	0.892	3.574
	Within (errors)	386,580.72	36	10,738.35			
	Total	4,506,231.91	47				
females							
pS8-Aβ42	Condition	148.05	1	148.05	0.32	0.575	7.396
	**Age**	357,497.08	5	71,499.42	154.507	**2.8e-23**	3.574
	Interaction	1593.83	5	318.77	0.689	0.635	3.574
	Within (errors)	16,659.28	36	462.76			
	Total	375,898.24	47				
Aβ42	**Condition**	3605.33	1	3605.33	6.954	**0.012**	7.396
	**Age**	383,645	5	76,729	147.986	**5.9e-23**	3.574
	Interaction	3418.1	5	683.62	1.318	0.278	3.574
	Within (errors)	18,665.55	36	518.49			
	Total	409,333.98	47				
isoD7-Aβ42	**Condition**	3918.66	1	3918.66	4.696	**0.037**	7.396
	**Age**	338,379.79	5	67,675.96	81.098	**1.4e-18**	3.574
	Interaction	5280.92	5	1056.18	1.266	0.299	3.574
	Within (errors)	30,041.99	36	834.5			
	Total	377,621.36	47				

*SS* Sum-of-squares, *DF* degrees of freedom, *MS* mean squares. Results of F-tests: F-value (F), *P*-value, F-critical value (F-crit). Significant estimates in bold

Numerous studies have previously shown that overexpression of Aβ significantly shortened *Drosophila* lifespan and locomotion function [13, 14]. Our results demonstrate that treatment with Aβ peptides induces a minor reduction in female lifespan, but more toxic Aβ42 form has no effect on female lifespan. Locomotor activity of Aβ overexpressed flies may demonstrate a progressive decrease during ageing, which is caused by Aβ peptide accumulation [30]. However, we did not observe overt effect of Aβ peptides on locomotion.

### Gene expression

We used the reverse transcription quantitative real-time polymerase chain reaction (RT-qPCR) to determine the effects of Aβ on the expression level of main AMP genes of *Drosophila*, namely: *Cecropin A1* (*CecA1*), *Defensin* (*Def*), *Drosocin* (*Dro*), *Drosomycin* (*Drs*), and *Metchnikowin* (*Mtk*). As compared with control variant, in males Aβ42 treatment induced 18-fold decrease of *CecA1* expression level, isoD7-Aβ42–5.3-fold increase of *Mtk* expression level, and pS8-Aβ42 increased expression level of *CecA1* (2-fold) and *Dro* (17.3-fold) (Fig. 1e). However, these changes did not affect lifespan and locomotor activity of males. We also found that treatment with Aβ peptides results in a 1.5- to 16.9-fold decrease in AMP genes expression relative to controls in females (Fig. 1f). The most significant negative effect on the level of AMP expression in females was induced by unmodified Aβ42 form.

Previous studies have shown the importance of oxidative stress in Aβ42 peptide toxicity in fly model [14]. We analyzed the expression level of redox-sensing enzyme gene *Peroxiredoxin 5* (*Prx5*), a negative regulator of the *Drosophila* immune response which is involved in trade-off between the antioxidant and immune functions [31]. Treatment with phosphorylated Aβ42 isoform, pS8-Aβ42 caused 6-fold elevation in the level of the *Prx5* in females, while other Aβ peptide did not affect the *Prx5* gene expression in males and females (Fig. 1e and f). We then investigated the expression level of transcription factor dFOXO, a positive regulator of the *Drosophila* AMPs [32] and found 4–5 fold decrease in the *dFOXO* mRNA levels in females (Fig. 1f). In addition, FOXO is a downstream component of insulin/IGF signaling pathway, which modulation may be associated with Aβ toxicity [33].

Since all variants of female treatment with Aβ peptides lead to similar changes in the level of AMP genes expression, but the effects on lifespan were observed in the Aβ42 and pS8-Aβ42 treated variants only, it can be concluded that changes in the relative levels of AMP genes expression were not sufficient to influence lifespan.

It should also be noted that our results are consistent with the previously described differences between males and females including the longer lifespan of females, the significantly higher locomotor activity in males, and gene expression levels as well as sex differences in the effects of pharmacological interventions on these parameters [34–36].

## Discussion

In this study the treatment with Aβ peptides causes a weak negative effect on the lifespan and slightly decreases the expression level of AMP genes of *Drosophila* females while locomotor activity is not affected. The activities of AMPs negative regulator *Prx5* and positive regulator *dFOXO* were increased and decreased, respectively. Probably a decrease of the production of endogenous AMPs may be due to the antimicrobial properties of exogenous Aβ peptides as it can have bactericidal properties.

The antimicrobial properties of Aβ peptides are well documented and believed to be caused by the ability to entrap pathogens and disrupt cell membranes with the mechanisms of oligomerization and fibrillization [7, 8]. The same mechanisms underlie the neurotoxic properties of Aβ peptides [4, 37]. The obtained results suggest that the chronic treatment with Aβ in *Drosophila* leads to prevalence of negative effects over positive ones.

The observed negative effect on lifespan is in disagreement with the finding where the extension of *Drosophila* lifespan was achieved by lowering the production of AMPs in the fat body beginning in the second half of lifespan [21]. This result could be explained by fundamental importance of temporal and tissue-specific control of AMP genes expression in lifespan regulation in contrast to the life-long and global influence of pharmacological treatment with endogenous Aβ. For example, age-associated inflammation in *Drosophila* fat body may repress AMPs production in the midgut and increase microbial proliferation, contribute to gut hyperplasia, leakage, and animal death [38]. In our experiments, Aβ peptides were received from food and they could affect AMP genes expression in the intestine with all negative consequences.

At the same time, it was shown that the negative effects of Aβ are not tissue-specific. The expression of the human Aβ42 peptide in adult *Drosophila* in a tissue- and time-controlled manner revealed that Aβ42 is also toxic in different non-neural cell types, including neurosecretory and epithelial cells [39]. The toxic effect may be associated with the Aβ-induced oxidative stress [14], as was evidenced by an increase in the expression level of *Prx5*.

It was also previously shown that the toxicity of Aβ overexpression in flies is associated with activation of the insulin/IGF signaling pathway [33]. Pro-longevity gene *dFOXO* is a component of insulin/IGF signaling pathway and positive regulator of AMPs expression [32]. The observed suppression of the activity of *dFOXO* by Aβ42 peptides can explain both a decrease of the biosynthesis of AMPs as well as a shortening of lifespan.

We also found differences in the effects of Aβ42 peptides in males and females. It is worth noting that sex differences of lifespan and healthspan effects as well as gene expression level in response to pharmacological treatments or genetic interventions are widespread in *Drosophila* and other model organisms [35, 36, 40]. We previously showed that activation of expression of AMP genes in response to entomopathogenic fungus demonstrate a sex-specific differences [41]. Both human studies and animal models revealed greater vulnerability to AD in females, while men are more likely to die from virtually all main causes of death [40, 42, 43]. This fact is consistent with our results on the greater susceptibility of females to Aβ42 peptides compared to males. The sex-specific and sex-biased effects of Aβ42 peptides may be related to patterns of gene expression, sex steroid hormones, differences in mitochondrial maintenance failure and other biological mechanisms [40, 43].

It is most difficult to explain the relationship between obtained effects and the isoform of amyloid. IsoD7-Aβ42 is known to be the most aggressive form of Aβ42, enforcing the formation of oligomers and peptide aggregates both in vitro and in mice model [29]. This isoform is much more neurotoxic than the native peptide. Contrary to Aβ42 and isoD7-Aβ42, phosphorylated peptide pS8-Aβ42 reduces plaque deposition in animals, inhibits zinc-dependent aggregation of amyloid, and prevents Na^+^, K^+^-ATPase inhibition [44]. At the same time pS8-Aβ42 has a much stronger tendency to spontaneous aggregation than Aβ42 and isoD7-Aβ42.

However, we found the opposite effects in flies. IsoD7-Aβ42 did not induce any effects. It is possible that isoD7-Aβ42 has a stronger antimicrobial property which compensates for its toxicity. The Aβ42 and pS8-Aβ42 affected lifespan despite their toxicity is much less than isoD7-Aβ42. This effects demonstrate that in the case of oral administration the amyloidogenic properties of the peptide do not play a crucial role.

## Conclusions

In this study we failed to confirm our suggestion about benefits of exogenous Aβ as a result of its antimicrobial activity. Rather, we revealed the weak negative effect of the oral administration of Aβ42 peptides on *Drosophila* lifespan. Treatment with Aβ42 and pS8-Aβ42 slightly decreased female’s median lifespan (by 4.5%). However, the effect on lifespan was not established for the more toxic peptide isoform isoD7-Aβ42. We failed to reveal overt effect of Aβ42 peptides on locomotion. The relative levels of AMP gene expression in male flies were largely unaffected by Aβ42 peptide treatment. Aβ42 peptide treatment induced slight decline in AMP genes expression in females regardless of the effects on lifespan. Thus, the oral intake of Aβ42 peptides does not appear to greatly affect fly aging or immunity.

## Materials and methods

### *Drosophila melanogaster* lines and maintenance conditions

Wild type *Canton-S* (#64349, Bloomington *Drosophila* Stock Center, USA) strain was used in all experiments. Control and experimental animals were maintained on nutrient medium containing 92 g cornmeal, 32.1 g yeast, 5.2 g agar, 136.9 g glucose, and 5 ml of propionic acid per 1 l. To maintain constant conditions (25 °C, 60% relative humidity, and 12 h/12 h light/dark cycle) the Binder KBF720-ICH (Binder, Germany) climate chamber was used.

### Treatment with beta-amyloid peptides

Synthetic peptides (purity > 98% checked by RP-HPLC) DAEFRHDSGYEVHHQKLVFFAEDVGSNKGAIIGLMVGGVVIA (Aβ42), DAEFRH [isoD]SGYEVHHQKLVFFAEDVGSNKGAIIGLMVGGVVIA (isoD7-Aβ42), and DAEFRH [pS] GYEVHHQKLVFFAEDVGSNKGAIIGLMVGGVVIA (pS8-Aβ42) were purchased from Biopeptide (San Diego, CA, USA). Peptides were treated with 10% NH_4_OH, dried and dissolved in water. Experimental adult flies were treated with synthetic peptides throughout their lifetime. For imago feeding, 30 μl of 20 μM peptide containing water solution was added to cover the media surface in each vial. To each control vial 30 μl of distilled water was added. Vials were dried under the fan for 1–2 h. Flies were flipped onto new media 2 times per week. One time per week the peptides were added to the experimental vials.

### Lifespan analysis

Newly eclosed flies were collected within 24 h and sorted by sex using light carbon dioxide anesthesia, and at density of 30 flies were housed in narrow vials (Genesee Scientific, USA). The number of dead flies was counted every day. For each experiment 2 replicates of 5 vials (150 flies) were analyzed. The median lifespan, maximum lifespan (age of 90% mortality), and the mortality rate doubling time (MRDT) were calculated.

### Locomotor activity analysis

The rate of spontaneous locomotor activity was measured using the LAM25 Locomotor Activity Monitor (TriKinetics Inc., USA). The data from 30 flies in 4 vials as replicates were collected during 24 h and represented as average daily locomotor activity per fly. Measurements were carried out every week, from the age of 3 to 9 weeks.

### Real-time quantitative reverse transcription PCR

Freshly emerged imagoes were collected within 24 h and treated with amyloid peptides for 10 days. The gene expression analyses were carried out using whole bodies of 20 males or 10 females per variant of experiment. Real-time quantitative reverse transcription-polymerase chain reaction (qRT-PCR) were used to measure the expression levels of genes related to immune response (*Cecropin A1* (*СecA1*), *Defensin* (*Def*), *Drosocin* (*Dro*), *Drosomycin* (*Drs*), *Metchnikowin* (*Mtk*), and *Peroxiredoxin 5* (*Prx5*)) and to insulin/IGF signaling pathway (*Drosophila* homolog of forkhead box O (FOXO) transcription factors (*dFOXO*)).

RNA was isolated by Aurum Total RNA mini kit (Bio-Rad, USA). To determine total RNA concentration was used Quant-iT RNA Assay Kit (Invitrogen, USA). Reverse transcription was performed using the iScript cDNA Synthesis Kit (Bio-Rad, USA). The mix for RT-PCR was prepared by iTaq Universal SYBR Green Supermix (Bio-Rad, USA) with primers listed in Table 4. The primer design was performed using QuantPrime [45]. The reaction was carried out on the CFX96 Real-Time PCR Detection System (Bio-Rad, USA) using the following parameters: one cycle of 95 °C for 30 s; 40 cycles of 95 °C for 10 s and 60 °C for 30 s. Expression levels of target genes was calculated relative to the expression of reference genes: *β-Tubulin56D* (*βTub56D*), *eukaryotic Elongation Factor 1α1* (*eEF1α1*), and *Ribosomal protein L32* (*RpL32*) using the Bio-Rad CFX Manager 3.1 (Bio-Rad, USA). Experiments were made in 3 independent biological replicates, with 3 technical replicates in each.

**Table 4:** List of primers used for real-time qRT-PCR analysis of gene expression

Gene	Forward	Reverse
*βTub56D*	5′-ggccaactgaacgctgatct-3’	5′-aagccgggcatgaagaagtg-3’
*eEF1α1*	5′-agggcaagaagtagctggtttgc-3’	5′-gctgctactactgcgtgttgttg-3’
*RpL32*	5′-acaggcccaagatcgtgaag-3’	5′-tgttgtcgatacccttgggc-3’
*Cecropin A1*	5′-tcgctcagacctcactgcaatatc-3’	5′-tgtccaatggtgatggccagaatg-3’
*Defensin*	5′--gttcttcgttctcgtggctatcg3’	5′-atccacatcggaaactggctgag-3’
*Drosocin*	5′-tcagttcgatttgtccacca-3’	5′-gatggcagcttgagtcaggt-3’
*Drosomycin*	5′-aagtacttgttcgccctcttcgc-3’	5′-acagggacccttgtatcttccg-3’
*Metchnikowin*	5′-tcgcccttcaatcctaaccaacc-3’	5′-acgacatcagcagtgtgaatttcc-3’
*Peroxiredoxin 5*	5′-ccgatgagctgaagtccaag-3’	5′-ttgccgttctccaccaccag-3’
*dFOXO*	5′-tagcagtgccggatggaagaac-3’	5′-accctcataaagcggttgtgcag-3’

### Statistical analysis

To assess the statistical significance of differences in median lifespan between control and experimental groups, the Fisher’s exact test was used [46]. The Boschloo’s (Wang-Allison) test was used to estimate the differences in the maximum lifespan (age of 90% mortality) [47]. Kaplan-Meier survival curves were plotted and statistical significance was assessed by the log-rank and Kolmogorov-Smirnov tests [48, 49]. To test the effects of Aβ peptides on lifespan, we used Cox regression models. Cox proportional hazards regression can evaluate the proportional effects of several risk factors on survival. Mortality rate can be explained by the proportional sum of risk factors. The procedure used the partial likelihood estimator to test the effects of covariates on the probability of survival at different ages. We considered Aβ peptide (treatment versus control) and vials (10 vials in each experimental variant) as covariates. Significance of locomotor activity at specific ages was calculated using Student’s t-test. The differences in locomotor activity levels among different ages or conditions (control and treatments) were calculated by using two-way analysis of variance (ANOVA). To compare the gene expression levels Student’s t-test was used. Statistical analysis of lifespan data and Cox proportional hazards analysis and two-way ANOVA test were performed using OASIS 2 online tool [50]. Statistical analysis of locomotor activity was done with STATISTICA 6.1 (StatSoft, USA). Real-time qRT-PCR data were analyzed using the Bio-Rad CFX Manager 3.1 (Bio-Rad, USA).

## Data Availability

Not applicable.

## Abbreviations

Aβ: Amyloid-β peptide
Aβ42: Beta-amyloid peptide 42
AD: Alzheimer’s disease
AMP: Antimicrobial peptide
ANOVA: Analysis of variance
IMD: Immune deficiency
isoD7-Aβ42: Aβ42 peptide with isomerized aspartic acid at the 7th residue
pS8-Aβ42: Aβ42 peptide with phosphorylated serine at the 8th residue
qRT-PCR: quantitative reverse transcription polymerase chain reaction
RP-HPLC: Reversed phase high-performance liquid chromatography
